# Comparative study of Japanese nationwide epidemiological studies of myasthenia gravis using datasets of 2006 and 2018

**DOI:** 10.1371/journal.pone.0334041

**Published:** 2025-10-09

**Authors:** Hiroaki Yoshikawa, Yumi Adachi, Yosikazu Nakamura, Nagato Kuriyama, Hiroyuki Murai, Yoshiko Nomura, Yasunari Sakai, Kazuo Iwasa, Yutaka Furukawa, Makoto Matsui, Satoshi Kuwabara, Taskforce of Research that Aims to Contribute to Improving the Medical Level of Intractable Diseases and Neuroimmune Patients’ Quality of Life

**Affiliations:** 1 Health Service Center, Kanazawa University, Kanazawa, Ishikawa, Japan; 2 Public Health Center, Utsunomiya City, Tochigi, Japan; 3 Shizuoka Graduate University of Public Health, Shizuoka City, Shizuoka, Japan; 4 Department of Neurology, School of Medicine, International University of Health and Welfare, Narita, Chiba, Japan; 5 Yoshiko Nomura Neurological Clinic for Children, Bunkyo-ku, Tokyo, Japan; 6 Department of Pediatrics, Graduate School of Medical Sciences, Kyushu University, Fukuoka, Japan; 7 Ishikawa Prefectural Nursing University, Kahoku, Ishikawa, Japan; 8 NHO Kanazawa Medical Center, Kanazawa, Japan; 9 Department of Neurology, Houju Memorial Hospital, Nomi, Ishikawa, Japan; 10 Department of Neurology, Graduate School of Medicine, Chiba University, Chiba City, Chiba, Japan; Transilvania University of Brasov: Universitatea Transilvania din Brasov, ROMANIA

## Abstract

**Objective:**

This study aimed to identify changes in the clinical presentation and treatment patterns of myasthenia gravis (MG) in Japan by comparing data from nationwide epidemiological surveys conducted in 2006 and 2018.

**Methods:**

We analyzed data from nationwide epidemiological surveys of MG performed across Japan in 2006 (n = 729) and 2018 (n = 1,357). Both surveys adhered to the Survey Manual of Study on Continuous Epidemiological Data Collection of Intractable Diseases established by the Ministry of Health, Labour, and Welfare. We compared the following variables between the two-time points: age of onset, gender distribution, initial symptoms, anti-acetylcholine receptor antibody (AChRAb) status, physiological test results (repetitive nerve stimulation test, edrophonium test), Myasthenia Gravis Foundation of America (MGFA) Clinical Classification, Myasthenia Gravis Activities of Daily Living Profile (MG-ADL) score, and treatment modalities.

**Results:**

Our analysis revealed a significantly older age of onset in the 2018 cohort compared to 2006. The MGFA Clinical Classification was higher in 2018, and bulbar weakness was more frequently reported as an initial symptom. Conversely, the occurrence of myasthenic crisis and current MG-ADL scores were lower in 2018. Regarding treatment, the utilization of tacrolimus, plasma exchange (PE), and intravenous immunoglobulin (IVIg) significantly increased between 2006 and 2018. In contrast, the rates of thymectomy and both maximum and current oral steroid dosages decreased during this period.

**Conclusion:**

These findings underscore notable shifts in the clinical characteristics and, particularly, the treatment strategies for MG in Japan over the twelve-year interval. This information is crucial for informing future patient care protocols and healthcare policy development in the management of MG. We emphasize the importance of conducting regular nationwide epidemiological surveys to monitor the evolving landscape of MG and its treatment continuously.

## Introduction

Myasthenia gravis (MG) is a chronic autoimmune disorder characterized by the disruption of neuromuscular transmission due to autoantibodies targeting postsynaptic molecules at the neuromuscular junction [1]. The foundational understanding of MG’s autoimmune basis emerged from the pioneering work of Patrick and Lindstrom, who demonstrated that rabbits developed muscle weakness following immunization with acetylcholine receptor (AChR) protein purified from electric eels [2]. Subsequently, Lindstrom et al. established the diagnostic significance of anti-AChR antibodies (AChRAb) in MG [3]. Further expanding our knowledge of MG heterogeneity, Hoch et al. identified autoantibodies against muscle-specific kinase (MuSK) in a substantial proportion (70%) of AChRAb-negative MG patients, marking MuSK as the second major pathogenic autoantigen [4]. While other potential autoimmune targets, such as low-density lipoprotein receptor-related protein 4 [5] and agrin [6], have been identified, their precise pathophysiological roles are still under investigation. Notably, the predominant IgG subclasses differ between the primary autoantibodies: AChRAb is primarily IgG1 and IgG3, whereas anti-MuSK antibodies (MuSKAb) are predominantly IgG4 [1]. Furthermore, thymic abnormalities, including thymoma or hyperplasia, are frequently observed in MG patients with AChRAb [7] but are rare in those with MuSKAb [8]. The co-occurrence of both AChRAb and MuSKAb in the same patient is exceedingly uncommon [9].

Beyond unraveling the intricate pathophysiology of MG, longitudinal epidemiological studies are crucial for monitoring the disease’s evolving landscape, including changes in patient demographics, clinical manifestations, and treatment approaches. Conducting reliable nationwide epidemiological surveys demands significant effort and resources. Recognizing the importance of such data, the Ministry of Health, Labour, and Welfare (MHLW) of Japan has undertaken four nationwide epidemiological studies of MG [10], conducted in 1973 [11], 1987 [12], 2006 [13], and 2018 [14]. Significantly, the latter two surveys (2006 and 2018) were designed using two steps. The first step aimed to estimate prevalence, and the second focused on clarifying clinical features and treatment patterns using a consistent survey methodology [15]. This study leverages the valuable datasets from these two comparable nationwide surveys to analyze the shifts in the clinical presentation of MG and its treatment modalities in Japan over twelve years.

## Materials and methods

### Study design

This study employed a cross-sectional comparative analysis of data from two nationwide epidemiological surveys of MG conducted in Japan in 2006 [13] and 2018 [14]. Both surveys adhered to the same survey manual, ‘Study on Continuous Epidemiological Data Collection and Intractable Diseases,’ issued by the MHLW of Japan [15] and utilized a two-step survey methodology.

However, it is important to note that the diagnostic criteria differed between 2006 and 2018. The 2006 diagnostic criteria were based on the presence of both subjective and objective symptoms, with confirmation through at least one of the following: repetitive nerve stimulation test (RNST), edrophonium test, or positive AChRAb (S1 Table). In contrast, the 2018 diagnostic criteria required the presence of subjective or objective symptoms and either a positive AChRAb or MuSKAb result. Additionally, the diagnosis was supported by abnormal findings on RNST or single-fiber electromyography (SFEMG) (S2 Table). The 2018 diagnostic criteria emphasized the underlying pathophysiology of MG, incorporating antibody testing as a primary diagnostic criterion.

The 2006 survey employed a two-step approach—the initial step aimed to estimate the prevalence and approximate number of MG patients nationwide [13]. Hospitals were randomly selected from a national registry, stratified by the number of beds. The sampling rates were approximately 5% for hospitals with 20–99 beds, increasing stepwise to 100% for hospitals with 500 or more beds. All university hospitals and clinics with many MG patients were also included. The initial survey asked for the number of MG patients who visited the hospitals between January 1 and December 31, 2005, which were sent to 5,426 departments, including 1,112 neurology/internal medicine, 980; surgery; 841; pediatrics, 808; ophthalmology, 776; otorhinolaryngology, 735; neurosurgery, and 174; cardiac surgery departments. In the subsequent step, questionnaires were distributed to the institutions that reported MG patients in the initial survey to collect detailed clinical information, including age at onset, gender, date of birth, family history, onset symptoms, symptoms at diagnosis, MG Foundation of America (MGFA) classification [16], MG activities of daily living profile (MG-ADL) score [17], edrophonium test, RNST, AChRAb, MuSKAb, thymic pathology, comorbidities, and therapies. The 2006 second step included patients who visited the participating hospitals between January 1 and December 31, 2006, regardless of their initial diagnosis date.

The 2018 survey mirrored the two-step methodology and the stratified random sampling of hospitals and departments used in the 2006 survey. 1,544; internal medicine, 719; neurology, 848; pediatrics, 1,091; surgery, 834; neurosurgery, 397; respiratory surgery, 476; cardiovascular surgery, 852; ophthalmology, and 784; otolaryngology.The questionnaires sent on March 30, 2018, requested the number of MG patients who visited their hospitals between January 1, 2017, and December 31, 2017. Furthermore, detailed clinical information was requested for patients diagnosed with MG between January 1, 2015, and December 31, 2017 (S3 Table). This restriction on the diagnosis period was implemented to ensure the accuracy of patient records. The study flow for the 2018 survey has been previously reported [14].

We systematically compared data between the 2006 and 2018 cohorts regarding gender, onset age, initial symptoms, clinical severities (MGFA Clinical Classification and MG-ADL score), laboratory tests (AChRAb, MuSKAb), physiological tests (RNST, edrophonium test), comorbidities, and treatments. The study center for the 2006 survey was the Department of Neurology, Kyushu University (led by HM). The 2018 survey was conducted at the Health Cervice Center, Kanazawa University (led by HY). We obtained the 2006 and 2018 datasets from the Research Taskforce that Aims to Improve the Medical Level of Intractable Diseases and Neuroimmune Patients’ Quality of Life. The dataset did not include information about participant identities.

### Protocol approvals and ethics statement

The Kanazawa University Medical Ethics Committee (2017−292) approved the study. This retrospective study was based on databases. The medical records from which the databases were created and the correspondence tables of patients were stored in the hospitals or departments that participated in this study. All data were fully anonymized before we accessed them, and the ethics committee waived the requirement for informed consent.

### Data analysis

We evaluated the distribution of continuous data using the Shapiro-Wilk test, which revealed a non-normal distribution. Continuous data were expressed as median (interquartile range [IQR]). We used the Wilcoxon-Mann-Whitney test (WMW) to compare the two continuous datasets. We used Fisher’s exact test to compare nominal data. Outliers were checked for, and missing values were considered. Variables with 50% or more missing data were excluded from the analysis. The remaining missing values were imputed using a low-rank matrix approximation method [18–21].

Multivariable analysis was performed using nominal logistic regression to assess the relationship between various characteristics and the outcome (i.e., the year 2018, with 2006 as the reference). Odds ratios (ORs) with 95% confidence intervals (CIs) were calculated to quantify the strength of association. Odds ratios were chosen as the primary measure of effect because they are appropriate for modeling dichotomous outcomes and provide a clear interpretation of the relative odds of the outcome occurring given the presence of an exposure or characteristic. All statistical analyses were conducted using JMP Pro 18 (SAS Institute Japan Ltd., Tokyo, Japan).

## Results

### Sample profiles

The initial datasets from the 2006 and 2018 nationwide epidemiological surveys contained 750 and 1,384 records, respectively. We excluded 48 cases where all four key diagnostic parameters (AChRAb, MuSKAb, Edrophonium test, and RNST) were either negative or had missing values. This resulted in a final sample size of 729 patients from the 2006 survey and 1,357 from the 2018 survey for subsequent analysis. The prevalence of specific comorbidities was very low: pure red cell aplasia (2006: n = 2, 2018: n = 0) and multiple sclerosis (2006: n = 0, 2018: n = 1). Due to their limited occurrence, these comorbidity categories were excluded from further comparative analysis. We identified and removed five outliers from the continuous data (MG-ADL: n = 1, AChRAb value: n = 1, MuSKAb value: n = 1, and oral steroid dose: n = 2) as their values were deemed to be outside the plausible biological range.

The distribution of missing values for all variables is detailed in S4 Table. Due to a high proportion of missing data (over 50%), the following factors were excluded from further analysis: MuSKAb status (positive/negative), MuSKAb titer, thymectomy method, thymic pathology, Masaoka classification for thymoma, and WHO classification for thymoma. Missing values for the remaining variables were imputed using the low-rank matrix approximation method, as described in the Data analysis subsection of the Materials and Methods.

### Baseline analysis

The baseline characteristics of the patient cohorts from the 2006 and 2018 surveys are summarized in Table 1 (for nominal variables) and Table 2 (for continuous variables). Table 2 presents explicitly the baseline analysis of continuous factors.

**Table 1:** Baseline analysis, nominal factors.

	2006 (n, %)	2018 (n, %)	odds ratio (95% CI)	P*
**Gender**				
**Male**	273, 37.5	631, 46.5	1.45 (1.21–1.75)	<0.0001^a^
**Initial symptom**				
**Blepharoptosis**	537, 73.7	991, 73.0	0.97 (0.79–1.19)	0.7954
**Diplopia**	353, 48.4	605, 44.6	0.86 (0.72–1.03)	0.0973
**Facial weakness**	33, 4.5	55, 4.1	0.89 (0.57–1.39)	0.6480
**Bulbar weakness**	97, 13.3	226, 16.7	1.30 (1.01–1.68)	0.0490
**Neck and extremities weakness**	153, 21.0	274, 20.2	0.95 (0.76–1.19)	0.6904
**Dyspnea**	12, 1.7	39, 2.9	1.77 (0.92 −3.40)	0.1013
**AChRAb**	617, 84.6	1,133, 83.9	0.94 (074–1.21)	0.6605
**Edrophonium test**	707, 97.0	1202, 95.2	0.62 (0.38–1.01)	0.0536
**RNST**	502, 68.9	898, 66.2	0.88 (0.73–1.07)	0.2219
**Crisis**	99, 13.6	103, 7.6	0.52 (0.39–0.70)	<0.0001
**Comorbidities**				
**Rheumatoid arthritis**	14, 1.9	26, 1.9	1.00 (0.52–1,92)	1.0000
**Hashimoto’s disease**	29, 4.0	72, 5.3	1.35 (0.87–2.10)	0.1996
**Graves’ disease**	38, 5.2	82, 6.0	1.17 (0.79–1.74)	0.4903
**SLE**	10, 1.4	6, 0.4	0.32 (0.12–0.88)	0.0318
**Family history of MG**	4, 0.6	14, 1.0	1.89 (0.62–5.76)	0.3257
**Treatment**				
**ChEI**	524, 71.9	1070, 78.9	1.46 (1.19–1.79)	0.0004
**Oral steroid**	533, 73.1	938, 69.1	0.82 (0.67–1.01)	0.0624
**tacrolimus**	145, 19.9	580, 42.7	3.01 (2.43–3.71)	<0.0001
**Steroid pulse therapy**	128, 17.6	284, 20.9	1.24 (0.99–1.57)	0.0737
**PE**	90, 12.4	1190, 87.7	50.59 (38.48–66.52)	<0.0001
**IVIg**	31, 4.3	288, 21.2	6.07 (4.14–8.89)	<0.0001
**Thymectomy**	500, 68.6	465, 34.3	0.24 (0.20–0.29)	<0.0001

*Fisher’s exact test.

MFGA: myasthenia gravis foundation of America, AChRAb: anti-acetylcholine receptor antibody, RNST: repetitive nerve stimulation test, SLE: systemic lupus erythematosus, ChEI: choline esterase inhibitor, PE: plasma exchange, IVIg: intravenous immunoglobulin.

**Table 2:** Baseline analysis, continuous factors.

	2006 (median [IQR])	2018 (median [IQR])	S	Z	P*
**Onset age**	47 (26–62)	60 (43–70)	608324	−11.6192	<0.0001
**MGFA Clinical Classification**	3 (2 −4)	3 (2–4)	742845	−1..41583	0.1568
**MG-ADL score initial**	5 (3–7)	5 (3–8)	745177	−1.18946	0.2343
**MG-ADL score current**	2.2 (1–4)	1.8 (0–3)	869902	8.39566	<0.0001
**AChRAb titer (nmol/L)**	24 (5.7–87)	28 (6.6–81)	753680	−0.53604	0.5919
**Oral steroid dose: maximum (mg/day)**	37 (30–45)	17 (10–25)	1079256	24.32262	<0.0001
**Oral steroid dose: current (mg/day)**	18 (15–25)	6 (5–10)	1131283	26.28252	<0.0001

*Wilcoxon-Mann-Whitney test (WMW).

MGFA: Myasthenia Gravis Foundation of America, MG-ADL: myasthenia gravis activities of daily living, AChRAb: anti-acetylcholine receptor antibody, RNST: repetitive nerve stimulation test, ChEI: choline esterase inhibitor, PE: plasma exchange, IVIg: intravenous immunoglobulin.

MGFA Clinical Classification was substituted in numbers as follows: 0 → 1, I → 2, IIa → 3, → IIb → 4, IIIa → 5, IIIb → 6, IVa → 7, IVb → 8, V → 9.

We performed multivariable analysis of factors that showed significant differences (P < 0.05) in Tables 1 and 2. The results of these comparisons are presented in Table 3.

**Table 3:** Multivariable analysis between 2006 and 2018.

	odds ratio (95% CI)	Chi-square	P*
**Gender**			
**Male**	1.20 (0.83–1.73)	0.97610373	0.3232
**Onset Age**	1.02 (1.01–1.03)	15.5497783	<0.0001
**Initial symptom**			
**Bulbar weakness**	2.07 (1.27–3.37)	8.80120714	0.0030
**MG-ADL score current**	0.86 (0.80–0.92)	17.5497783	<0.0001
**Crisis**	0.43 (0.25–0.76)	8.92345038	0.0028
**Comorbidities**			
**SLE**	0.66 (0.14–3.04)	0.29656227	0.5860
**Treatment**			
**ChEI**	0.92 (0.61–1.39)	0.14810931	0.7003
**tacrolimus**	2.82 (1.87–4.27)	25.3876912	<0.0001
**PE**	41.79 (28.53–61.21)	505.265444	<0.0001
**IVIg**	24.93 (13.50–46.01)	138.707479	<0.0001
**Thymectomy**	0.42 (0.29–0.62)	19.6576942	<0.0001
**Oral steroid dose, maximum (mg/day)**	0.97 (0.96–0.98)	20.7855932	<0.0001
**Oral steroid dose, current (mg/day)**	0.87 (0.85–0.89)	114.38142	<0.0001

*Nominal logistic analysis. The whole model was significant (P < 0.0001).

MFGA: Myasthenia Gravis Foundation of America, AChRAb: anti-acetylcholine receptor antibody, RNST: repetitive nerve stimulation test, SLE: systemic lupus erythematosus, ChEI: choline esterase inhibitor, PE: plasma exchange, IVIg: intravenous immunoglobulin.

The multivariable analysis revealed that thymectomy had an adjusted odds ratio of 0.42 (95% CI: 0.29–0.62, p < 0.0001). This suggests that individuals with thymectomy had 0.42 times the odds (or a 58% lower odds) of being in the 2018 cohort compared to those without thymectomy, when controlling for other factors. For tacrolimus treatment, the adjusted odds ratio was 2.82 (95% CI: 1.87–4.27, p < 0.0001). This indicates that individuals with tacrolimus had 2.82 times higher odds of being in the 2018 cohort compared to those without tacrolimus, after adjusting for other variables in the model.

### Key findings

The comparative analysis revealed significant changes in the 2018 cohort compared to the 2006 cohort:

1. Increased: Age of onset and the proportion of patients presenting with bulbar weakness as an initial symptom.
2. Decreased: Occurrence of myasthenic crisis and current MG-ADL scores.
3. Increased Treatment Usage: Tacrolimus, plasma exchange (PE), and intravenous immunoglobulin (IVIg) therapies.
4. Decreased Treatment Usage: Thymectomy rates and both maximum and current oral steroid dosages.

### Visual representation of key findings

**Fig 1:**
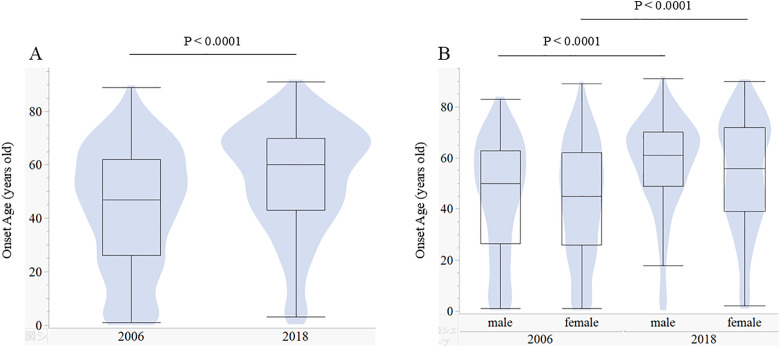
The onset age of 2006 and 2018 for total male and female patients. Overlapping presentation of a box and violin plots for onset ages by study years. The areas of the violin plots are proportional to the number of patients. The horizontal line within the box represents the median value. The box ends define the 25th and 75th quantiles, expressed as the first and third quartiles. The lines that extend from the box end are whiskers. The whiskers extend from the ends of the box to the outermost data point that falls within these distances: first quartile–1.5 x (interquartile range), 3rd quartile+1.5 x (interquartile range). A. total patients, B. by gender.

Fig 1 illustrates the distribution of onset ages in the 2006 and 2018 surveys, stratified by gender and for the total patient population.

**Fig 2:**
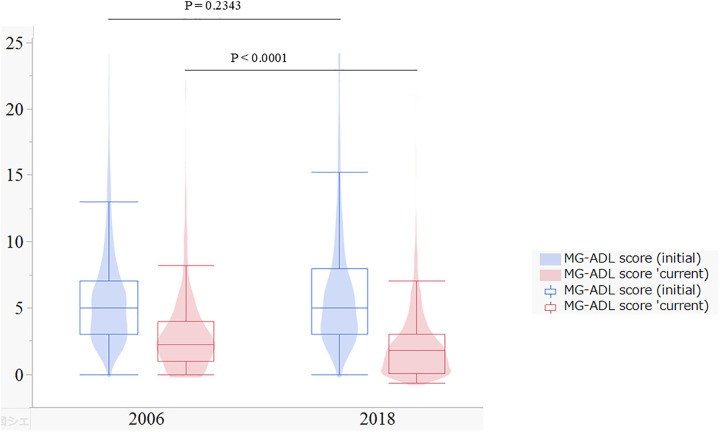
MG-ADL score at initial evaluation and current by survey years. Overlapping presentation of a box and violin plots for MG-ADL score by study years.

Fig 2 shows the distributions of MG-ADL scores at initial evaluation and at the time of the survey (current status) for both the 2006 and 2018 cohorts.

**Fig 3:**
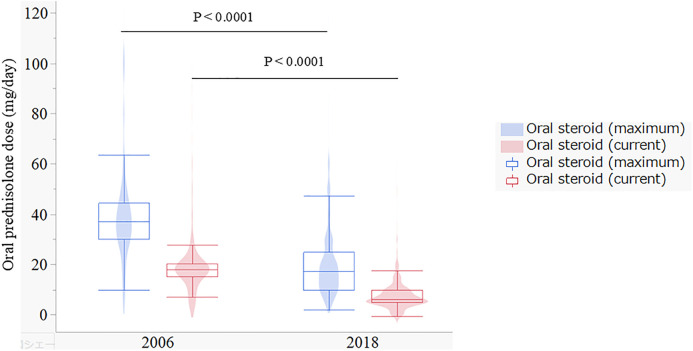
Oral steroid doses at maximum and current by the survey years.. Overlapping presentation of a box and violin plots for oral steroid doses (maximum and current) by survey years.

Fig 3 presents the maximum and current oral steroid doses reported in the 2006 and 2018 surveys.

## Discussion

Our study reveals significant shifts in MG’s clinical presentation and treatment landscape in Japan between 2006 and 2018. These findings underscore crucial trends in MG management and offer valuable insights for shaping future patient care strategies and healthcare policies within Japan.

MG, a rare autoimmune neuromuscular disorder, has been the subject of nationwide epidemiological studies across the globe, including Norway and the Netherlands [22], Austria [23], Sweden [24,25], Hungary [26], Australia [27,28], the Faroe Islands [29], Taiwan [30], Israel [31], South Africa [32], Japan [13,14], Korea [33], and Latvia [34]. While these studies primarily reported incidence and prevalence within their respective regions, a global trend of increasing MG incidence and prevalence has been noted [35,36]. This rise is likely multifactorial, potentially stemming from improved disease recognition and diagnostic capabilities [37], increased longevity and an aging global population [35], possible viral triggers [37], and even socioeconomic factors [38].

Of particular interest is the observed increase in MG onset among older individuals. The growing tendency towards elderly-onset MG (over 60 years) and the attenuation of female predominance was reported as early as 2001 [39] and have been corroborated by nationwide studies in Austria [23] and Israel [31], as well as a German retrospective study [40]. Our finding of significantly higher onset age in the 2018 Japanese cohort aligns with this global trend, providing crucial insights into the evolving epidemiology of MG in an aging society. However, it is essential to acknowledge the inherent limitations of multi-center epidemiological studies, such as variations in diagnostic criteria and data collection methodologies across different regions.

Despite these challenges, reports detailing repeated nationwide surveys, such as ours, remain scarce. Therefore, this study offers valuable longitudinal data for healthcare professionals and policymakers. Furthermore, the significant changes we observed in MG treatments within 12 years likely reflect the influence of updated MG treatment guidelines, notably the revisions made in 2014 [41].

Interestingly, while previous Japanese nationwide studies (1973, 1987, 2006) reported a female predominance in MG [10], our 2018 study indicated a near-even female-to-male ratio [14], a trend also observed in Israel [31]. However, our direct comparison between 2006 and 2018 did not reveal a statistically significant shift in gender prevalence.

MG treatment patterns have also been evolving in other parts of the world. A South Korean study demonstrated a gradual decrease in steroid and azathioprine use between 2010 and 2018, coupled with increased tacrolimus utilization [33]. Conversely, studies from Poland [42] and Australia [28] reported a higher prevalence of steroid treatment. The established safety and efficacy of thymectomy for AChRAb-positive non-thymomatous MG patients, as evidenced by the MGTX study [43,44], and the recognized equivalence of video-assisted thoracic surgery to open thymectomy [45], have led to recommendations from the American Academy of Neurology (AAN) to discuss thymectomy with such patients [46]. A recent US report showed a significant annual increase in thymectomy procedures [47]. However, our findings reveal a significant decrease in thymectomy rates in Japan in 2018 (34.3%) compared to 2006 (68.6%), suggesting potential differences in treatment paradigms or patient selection for surgery in Japan.

Another striking change in our study was the increased utilization of tacrolimus, PE, and IVIg in 2018. This trend coincided with a decrease in both maximum and current oral steroid dosages, potentially indicating a shift towards steroid-sparing immunosuppressive strategies and increased use of acute immunotherapies. While the MGTX study utilized alternate-day steroid administration with good tolerability [43,44], our survey did not capture data on adverse events, quality of life, or treatment costs, all of which are crucial factors for comprehensive healthcare planning.

A key limitation of our study is the difference in diagnostic criteria employed in the 2006 and 2018 surveys. This evolution in diagnostic approaches could have influenced patient classification and perceived disease severity. We also did not consider demographic factors between 2006 and 2018. Furthermore, we did not assess the economic implications of the observed treatment changes. The substantial financial burden associated with MG therapy has been documented in other studies [48,49].

Additionally, our study did not systematically evaluate comorbidities beyond autoimmune diseases or the adverse effects of treatments. We also lacked fundamental patient data such as height, weight, blood pressure, laboratory results, and detailed past medical histories. Future epidemiological surveys should aim to address these gaps.

Moving forward, it will be crucial to consider the age demographics of the Japanese population in 2006 and 2018 to understand better the reasons behind the observed shift towards older-onset MG. To ensure consistency and accuracy in future surveys, adopting the methodology of the 2018 study is recommended. While updating diagnostic criteria based on advancements in understanding MG pathophysiology is essential, maintaining the integrity of historical data collection methods also warrants careful consideration. Future data collection should also aim to limit the inclusion of newly diagnosed cases to a defined recent period (e.g., three years, including the survey year) to enhance data accuracy.

A cautious interpretation of our findings is warranted, considering that our study population was predominantly Japanese, and genetic predispositions may differ from other ethnic groups. While genetic analysis alongside epidemiological surveys could further elucidate MG pathophysiology, the observed changes in clinical presentation and treatment over the past decade in Japan appear to mirror trends seen in other Western and Asian countries, suggesting a broader shift in MG epidemiology. To gain more robust causal insights into therapeutic trends and disease progression, prospective longitudinal cohort studies following patients from diagnosis are essential.

In conclusion, our study highlights a significant increase in elderly-onset MG in Japan between 2006 and 2018. Regarding treatment, the increased use of tacrolimus, PE, and IVIg, coupled with a decrease in thymectomy rates and oral steroid dosages, are noteworthy findings. Future periodic nationwide epidemiological studies, complemented by multifactorial analyses of patient conditions on a global scale, are necessary to optimize the management of MG patients and inform evidence-based medical policies.

## Supporting information

S1 Table Diagnostic criteria for MG in 2006 study. (DOCX)

S2 Table Diagnostic criteria for MG in 2018 study. (DOCX)

S3 Table Patient record for following survey in 2018 study. (DOCX)

S4 Table Missing values of factors. (DOCX)

S1 File Minimal dataset for the study. (XLSX)

S2 File Research protocol original. (PDF)

S3 File Research protocol English. (PDF)

S4 File STROBE checklist. (DOCX)
