# Supplementary material for: Comparative study of Japanese nationwide epidemiological studies of myasthenia gravis using datasets of 2006 and 2018
Source: PLoS One. 2025 Oct 9;20(10):e0334041. doi: 10.1371/journal.pone.0334041 (PMC12510604; doi:10.1371/journal.pone.0334041)
Supplement: S1 Table — (DOCX) [file pone.0334041.s001.docx]

S1 Table. Diagnostic criteria for MG in 2006 study

| 1 | One or more of the following subjective findings was present | | | | |
| --- | --- | --- | --- | --- | --- |
|  | 1 | Ptosis | | | |
|  | 2 | Doble vision | | | |
|  | 3 | Limb weakness | | | |
|  | 4 | Swallowing difficulty |  |  |  |
|  | 5 | Speech difficulty |  |  |  |
|  | 6 | Breathing difficulty | | | |
| 2 | One or more of the following objective findings was present | | | | |
|  | 1 | Ptosis | | | |
|  | 2 | Ophthalmoplegia | | | |
|  | 3 | Facial weakness | | | |
|  | 4 | Neck weakness |  |  |  |
|  | 5 | Limb and/or truncal weakness | | | |
|  | 6 | Dysphagia | | | |
|  | 7 | Dysarthria | | | |
|  | 8 | Dyspnea | | | |
| 3 | Diurnal fluctuation and/or easy fatigability were present | | | | |
| 4 | At least one of the following tests was positive | | | | |
|  | 1 | Edrophonium test | | | |
|  | 2 | Decremental response on repetitive nerve stimulation or anti AChR antibody | | | |
| 5 |  | Other conditions were excluded | | | |

AChR: acetylcholine receptor

S1 Table was constructed by Murai, H. et al. 2011 [10].
