# Supplementary material for: Comparative study of Japanese nationwide epidemiological studies of myasthenia gravis using datasets of 2006 and 2018
Source: PLoS One. 2025 Oct 9;20(10):e0334041. doi: 10.1371/journal.pone.0334041 (PMC12510604; doi:10.1371/journal.pone.0334041)
Supplement: S2 Table — (DOCX) [file pone.0334041.s002.docx]

S2 Table. Diagnostic criteria for MG in 2018 study

| 1 | Having the following subjective and objective symptoms, accompanied by easy fatigue and circadian fluctuations | | | | |
| --- | --- | --- | --- | --- | --- |
|  | 1 | Blepharoptosis | | | |
|  | 2 | Eye movement restriction | | | |
|  | 3 | Facial muscle weakness | | | |
|  | 4 | Dysarthria |  |  |  |
|  | 5 | Dysphagia |  |  |  |
|  | 6 | Masticatory disorders | | | |
|  | 7 | Neck muscle weakness | | | |
|  | 8 | Muscle weakness in extremities and trunk | | | |
|  | 9 | Dyspnea |  |  |  |
| 2 | Positive: one of the following autoantibodies | | | | |
|  | 1 | Anti-AChR antibody |  |  |  |
|  | 2 | Anti-MuSK antibody |  |  |  |
| 3 | Physiological findings indicating neuromuscular junction disorder by the following tests | | | | |
|  | 1 | Low-frequency repetitive nerve stimulation evoked electromyogram | | | |
|  | 2 | Edrophonium test (Evaluate using objective indices such as eye movement disorders and low-frequency repetitive stimulation-induced electromyogram) | | | |
|  | 3 | Single fiber EMG (SFEMG) | | | |
| 4 | Differential diagnosis | | | | |
|  |  | Diseases that cause ophthalmoplegia, limb muscle weakness, and dysphagia / respiratory disorders are all differentiation candidates. | | | |
|  |  | Lambert-Eaton myasthenia syndrome, Muscular dystrophy (Becker type, Limb-girdle type, Facio-scapulohumeral type), Polymyositis, Periodic paralysis, Hyperthyroidism, Mitochondrial encephalomyopathy, Progressive ophthalmoplegia, Guillain-Barre syndrome, Polyneuritis, Oculomotor nerve palsy, Tolosa-Hunt syndrome, Brainstem tumors and vascular disorders, Brainstem encephalitis, Herpes simplex and another viral encephalitis, Basal meningitis, Temporal arteritis, Wernicke's encephalopathy, Lee's encephalopathy, Diabetic extraocular palsy, Vasculitis, Neuro-Behcet's disease, Sarcoidosis, Multiple sclerosis, acute disseminated encephalomyelitis, Fisher syndrome, Congenital myasthenia syndrome, Congenital myopathy, Myotony, Blepharospasm, Apraxia of the eyelid, Amyotrophic lateral sclerosis, Eyelid skin laxity, Botulism | | | |
| Diagnosis criterion | | | | | |
| A | At least one of [1] and one of [2] are satisfied | | | | |
| B | At least one of [1] and any of [3], plus exclude all diseases listed in [4] | | | | |

AChR: acetylcholine receptor, MuSK: muscle-specific kinas
