# Supplementary material for: Comparative study of Japanese nationwide epidemiological studies of myasthenia gravis using datasets of 2006 and 2018
Source: PLoS One. 2025 Oct 9;20(10):e0334041. doi: 10.1371/journal.pone.0334041 (PMC12510604; doi:10.1371/journal.pone.0334041)
Supplement: S3 Table — (DOCX) [file pone.0334041.s003.docx]

S3 Table. Patient record for following survey in 2018 study

| Patient number【 】 | | | | | |
| --- | --- | --- | --- | --- | --- |
| Initial ＿：＿ (optional) | | Gender: 1. male 2. female | Birthday  Age: year-old | | |
| Residence | | At birt：（　　　　　　　　　　　　） | | | Current：（　　　　　　　　　　　　） |
| Estimated onset year and month: | | | | First consultation date: | |
| Confirmed diagnosis date: | | | | Medical institution diagnosed: | |
| Clinical symptoms | | | | | |
| Initial symptoms: 1．Blepharoptosis, 2．Eye movement disorder, 3．Facial weakness, 4．Dysarthria, 5．Dysphagia, 6. Masticatory muscle weakness, 7. Neck muscle weakness, 8. Extremities and trunk weakness, 9. Dyspnea, 10. Others（　　　　　　　　　　　　　　） | | | | | |
| MGFA clinical classification（the most severe）（Table 1） 1. 0 2. I 3. IIa 4. IIb 5. IIIa 6. IIIb 7 IVa 8. IVb 9. V | | | | | |
| MGFA clinical classification（at present）（Table 1） 1. 0 2. I 3. IIa 4. IIb 5. IIIa 6. IIIb 7 IVa 8. IVb 9. V | | | | | |
| MG-ADL score（the most severe） Total ( ), unknown, (at present) Total (　　　), unknown | | | | | |
| Current living situation 1．Working, 2．Go to school, 3．Domestic labor, 4．Home care, 5．Hospitalization, 6．Admission, 7．Others（　　） | | | | | |
| Laboratory findings（highest value） | | | | | |
| Anti-AChR antibody 1. Positive ( nmol/L), 2. Negative, 3. Not tested, 4. Unknown | | | | | |
| Anti-MuSK antibody 1. Positive ( nmol/L), 2. Negative, 3. Not tested, 4. Unknown | | | | | |
| Waning by low-frequency repetitive nerve stimulation 1. Positive, 2. Negative, 3. Not tested, 4. Unknown | | | | | |
| Abnormal findings by single-fiber electromyography 1. Positive, 2. Negative, 3. Not tested, 4. Unknown | | | | | |
| Edrophonium test 1. Positive, 2. Negative, 3. Not tested, 4. Unknown | | | | | |
| Information for thymus | | | | | |
| Radiological diagnosis by CT MRI) 1. Tumor, 2. Hyperplasia, 3. Normal, 4. Others ( ), 5. Unknown | | | | | |
| Thymectomy 1. Yes, 2. No, 3. Unknown | | | | | |
| The surgical approach 1. Transsternal, （a. Simple, b. Extended）, 2. Video-assisted, 3. Others ( ), 5. Unknown | | | | | |
| Radiation therapy 1. Yes（a. pre-operational, b. post-operational）, 2. No, 3. Unknown | | | | | |
| Chemotherapy 1. Yes, 2. No, 3. Unknown | | | | | |
| Pathologic finding 1. Thymoma, 2. Hyperplasia, 3. Involuted（normal）, 4. others（ ）, 5. unknown | | | | | |
| Masaoka's classification 1. Stage I, 2. Stage II, 3. Stage III, 4. Stage IVa, 5. Stage IVb, 6. Unknown | | | | | |
| WHO classification of thymoma 1. A, 2. AB, 3. B1, 4. B2, 5. B3, 6. Unknown | | | | | |
| Past history・complication 〇: Yes, ×: No, △: unknown） | | | | | |
| Autoimmune disease: 1. Rheumatoid arthritis ( ), 2. Hashimoto disease ( ), 3. Graves’ disease ( ), 4. Systemic lupus erythematosus ( ), 5. Pure red cell aplasia ( ), 6. Multiple sclerosis ( ), 7. Other autoimmune disease ( ) | | | | | |
| Therapies received | | | | | |
| 1. Cholinesterase inhibitor 1. Yes, 2. No | | | | | |
| 2. Steroid（a. prednisolone，b. others: ）, Maximum dose（ mg/day）, current dose（ mg/day）（average dose） | | | | | |
| 3. Immunosuppressant（a. tacrolimus, b. ciclosporin, c. others: ），current dose（ mg/day） | | | | | |
| 4. steroid pulse therapy, 5 Blood purification（a. Simple plasma exchange, b. immunoadsorption, c. double filtration）, 6. Intravenous immunoglobulin (IVIg) | | | | | |
| Crisis 1. Yes（a. Post-operational, b. post-infection, c. others: ）, 2. No, 3. Unknown | | | | | |
| Family-onset myasthenia gravis: 1. Yes (relationship: ), 2. No, 3. Unknown | | | | | |
| Other autoimmune diseases: 1. Yes (disease: relationship ), 2. No, 3. Unknown | | | | | |
| Prognosis | Length of hospital stay: ___________ months  Final condition: after _______ months of onset, modified Rankin Scale (MRS): 0, 1, 2, 3, 4, 5, 6  Exacerbation: 1. Yes, 2. No, Death: 1. Yes, 2. No, Cause of death: 1. Myasthenia gravis, 2. Others ( ) | | | | |

MGFA: Myasthenia Gravis Foundation of America, CT: computed tomography, MRI: magnetic resonance imaging
