# Supplementary material for: Comparative study of Japanese nationwide epidemiological studies of myasthenia gravis using datasets of 2006 and 2018
Source: PLoS One. 2025 Oct 9;20(10):e0334041. doi: 10.1371/journal.pone.0334041 (PMC12510604; doi:10.1371/journal.pone.0334041)
Supplement: S4 Table — (DOCX) [file pone.0334041.s004.docx]

**S4 Table. Missing Values of factos**

| **Factors** | **Property** | **Missing value**  **(n, %)** |
| --- | --- | --- |
| 1-2006 2-2018 | nominal | 0, 0 |
| 1. male 2. female | nominal | 5, 0.24 |
| Onset Age | continuous | 0, 0 |
| MGFA clinical classification: 1-0, I-2, Iia-3, Iib-4, IIIa-5, IIIb-6, IVa-7, IVb-8, V-9 | Ordinal | 20, 0.96 |
| MG-ADL score at first visit | continuous | 214, 10.26 |
| MG-ADL score current | continuous | 411, 19.70 |
| AChRAb:１-positive, ２-negative | nominal | 6, 0.29 |
| AChRAb titer (nmol/L) | continuous | 365, 17.50 |
| MuSKAb:１-positive, ２-negative | nominal | 1570, 75.26 |
| MUSKAb titer (nomol/L) | continuous | 2037, 97.65 |
| Family history of MG | nominal | 208, 9.97 |
| Edrophonium test（１-positive, ２-negative） | nominal | 476, 22.82 |
| Repetitive nerve stimulation test (RNST): 1-positive, 2-negative | nominal | 460, 22.05 |
| Initial symptom (blepharoptosis): 1-yes, 2-no | nominal | 0, 0 |
| Initial symptom (diplopia): 1-yes, 2-no | nominal | 0, 0 |
| Initial symptom (facial weakness): 1-yes, 2-no | nominal | 0, 0 |
| Initial symptom (bulbar palsy): 1-yes, 2-no | nominal | 1, 0.05 |
| Initial symptom (neck, extremities weakness): 1-yes, 2-no | nominal | 0, 0 |
| Initial symptom (dyspnea): 1-yes, 2-no | nominal | 0, 0 |
| Thymectomy: 1-yes, 2-no | nominal | 191, 9.16 |
| Thymectomy method: 1-open surgery 2-VATS | nominal | 1265, 60.64 |
| Thymic pathology: 1-Thymoma, 2-hyperplasia, 3-normal | nominal | 1244, 59.64 |
| Masaoka classification: 1-Stage I, 2-Stage II, 3-Stage III, 4-Stage IVa, 5-Stage IVb | nominal | 1660, 79.58 |
| WHO classification of thymoma: 1-A, 2-AB, 3-B1, 4-B2, 5-B3 | nominal | 1752, 83.99 |
| Rheumatoid arthritis: 1-yes, 2-no | nominal | 103, 4.94 |
| Hashimoto disease: 1-yes, 2-no | nominal | 115, 5.51 |
| Graves disease: 1-yes, 2-no | nominal | 110, 5.27 |
| Systemic lupus erythematosus (SLE): 1-yes 2-no | nominal | 107, 5.13 |
| Pure rec cell aplasia (PRCA): 1-yes 2-no | nominal | 100, 4.79 |
| Multiple sclerosis (MS) 1-yes 2-no | nominal | 100, 4.79 |
| Choline esterase inhibitor (ChEI): 1-yes, 2-no | nominal | 11, 0.53 |
| Oral steroid: 1-yes, 2-no | nominal | 13, 0.62 |
| Oral steroid dose: maximum | continuous | 844, 40.46 |
| Oral steroid dose: current | continuous | 942, 45.16 |
| Tacrolimus: 1-yes 2-no | nominal | 0, 0 |
| Steroid pulse therapy: 1-yes 2-no | nominal | 11, 0.58 |
| Plasma exchange (PE): 1-yes 2-no | nominal | 17, 4.55 |
| Intravenous immunoglobulin therapy (IVIg): 1-yes 2-no | nominal | 12, 0.58 |
| Crisis: 1-yes 2-no | nominal | 95, 4.55 |
