# Supplementary material for: Comparative study of Japanese nationwide epidemiological studies of myasthenia gravis using datasets of 2006 and 2018
Source: PLoS One. 2025 Oct 9;20(10):e0334041. doi: 10.1371/journal.pone.0334041 (PMC12510604; doi:10.1371/journal.pone.0334041)
Supplement: S2 File — (PDF) [file pone.0334041.s006.pdf]

研究計画書

１．課題名：

重症筋無力症及びランバート・イートン筋無力症の全国疫学調査

２．研究の概要・目的・意義

我国における難病対策は、1972 年制定の難病対策要綱の制定以来、難治性疾患克服研究事業として様々な疾患において進められてきた。免疫性神経疾患においても、免疫性神経疾患調査研究班において病態・病因の解明、治療方法の開発等が行われた。重症筋無力症（MG）は、当初より特定疾患として調査・研究が行われてきたが、現在は指定難病の一つとして引き続き病因の解明や治療方法の検討がされている。MG に関する全国疫学調査は 1973 年、1987 年、2005 年に実施された。前回の調査[1]から 10 年以上が経過した今、再度、我国の MG 患者の現状を把握することは意味があると思われる。また、MG 診断基準は 2015 年度に難治性疾患等政策研究事業（難治性疾患政策研究事業）（研究課題名）「エビデンスに基づいた神経免疫疾患の早期診断基準・重症度分類・治療アルゴリズムの確立」（略称「エビデンス班」）の研究として、自己抗体測定の進歩を踏まえて改訂されている[2]。

一方、神経筋接合部の免疫性神経疾患であるランバート・イートン筋無力症候群（LEMS）に関しては、患者数が推計できる全国疫学調査が実施された経緯はなく、指定難病の対象にもなっていない。LEMS の診断基準は、エビデンス班の研究成果として 2015 年に定められた[3]。

新しい診断基準を用いて、MG と LEMS の全国疫学調査を行い、我国におけるこの 2 疾患の疫学的理解を深めるとともに、今後のエビデンスに基づいた医療の推進に役立てることは重要な課題である。特に LEMS は、全国疫学調査により患者数の推定を行うことが指定難病として認定されるために必要である。なお、今回の研究計画は難治性疾患等政策研究事業（難治性疾患政策研究事業）（研究課題名）「神経免疫疾患のエビデンスによる診断基準・重症度分類・ガイドラインの妥当性と患者 QOL の検証 」（略称「新エビデンス班」）の研究事業の一つとして実施される。

なお、2006 年に今回と同様の方法で、重症筋無力症の全国疫学調査が実施されている（研究責任者：国際医療福祉大学医学部神経内科　村井弘之）。2006 年の匿名化されたデータの供与を村井弘之（今回の研究の共同研究者）から受け、今回（2018）の調査結果との比較検討も行う。

３．研究の科学的合理性と根拠

厚生労働科学研究費補助金 難治性疾患等政策研究事業（難治性疾患政策研究事業）「難治性疾患の継続的な疫学データの収集・解析に関する研究」（代表者　中村好一）が作成した「難病の患者数と臨床疫学像把握のための全国疫学調査マニュアル第３版」（2017 年１月）[4]（添付資料１）に従って、実施計画を立案した。

４．研究対象者の選定方針

（１）適格基準

「難病の患者数と臨床疫学像把握のための全国疫学調査マニュアル第 3 版」に従い、全国の医療施設に郵送により依頼文書と調査票を送り、回答をもとに集計をする。

a)　調査対象とする診療科

厚生労働省の医療施設動態調査票に基づき、次の診療科を調査対象にした。

神経内科、内科、小児科、呼吸器外科、心臓血管外科、外科、脳神経外科、眼科、耳鼻いんこう科

b)　特別階層病院

とくに患者が集中すると考えられる特別な病院として、以下の病院を設定した。

1. 独立行政法人　国立病院機構 北海道医療センター
2. 公益社団法人　総合花巻病院
3. 神経内科 千葉
4. 野村芳子 小児神経学クリニック
5. 独立行政法人　国立病院機構　宇多野病院
6. 独立行政法人　国立病院機構　長崎川棚医療センター

（２）除外基準

設定なし

５．目標数と研究実施期間

（１）目標数

1 次調査：7,545 機関（MG と LEMS を同時に調査する）

2 次調査：MG=2,000 機関、LEMS=200 機関（予定）

調査対象患者は、以下の条件を満たすものとする。

1 次調査

1. MG　　診断基準を満たし、2017 年 1 年間（2017 年 1 月 1 日～2017 年 12 月 31 日）に受診した患者（初診・再診を問わず全例）
2. LEMS　診断基準を満たし、2017 年 1 年間（2017 年 1 月 1 日～2017 年 12 月 31 日）に受診した患者（初診・再診を問わず全例）

2 次調査

1. MG　　1 次調査で該当する患者のうち、最近 3 年間（2015 年 1 月 1 日～2017 年 12 月 31 日）に確定診断された患者
2. LEMS　1 次調査で該当する患者のすべて

（２）研究実施期間

研究実施期間：承認日～　2023 年 3 月 31 日（西暦）
研究対象期間：2017 年 1 月 1 日～2017 年 12 月 31 日
解析期間　　：承認日～2027 年 3 月 31 日

６．研究方法

依頼状並びに調査票を抽出した医療機関に郵送し、調査を依頼する。回答は郵送として、集計の後、解析を行う。1 次調査では推計患者数を算定し、2 次調査では患者臨床像を明らかにするための調査を行う。
なお、1 次調査では MG ならびに LEMS 患者に関する調査を同時に行う。2 次調査では、MG 患者と LEMS 患者を別々に調査する。調査に必要な用紙を以下に列挙、参考資料として添付する。

1 次調査発送書類　　　　　発送時期：2018 年 3 月

1. 1 次調査依頼状　　　　　（添付資料 2）
2. 1 次調査再依頼状　　　　（添付資料 3）
3. MG 診断基準　　　　　　（添付資料 4）
4. LEMS 診断基準　　　　　（添付資料 5）
5. 1 次調査票（葉書）　　　（添付資料 6）

1 次調査票回収後、患者無の場合

礼状　　　　　　　　　　　（添付資料 7）

2 次調査発送書類　　　　　発送時期：2018 年 9 月

a) MG 患者がいる施設

1. MG 2 次調査依頼状　（添付資料 8）
2. MG 2 次調査個人票　（添付資料 9）
3. 対応表（MG）　（添付資料 10）
4. MG 診断基準　（添付資料 4）
5. 資料 1（MGFA 分類）　（添付資料 11）
6. 資料 2（MG-ADL scale）　（添付資料 12）
7. 資料 3（正岡分類）　（添付資料 13）
8. 資料 4（mRS）　（添付資料 14）
9. 情報公開文書　（添付資料 15）

b) LEMS 患者がいる施設

1. LEMS 2 次調査依頼状　（添付資料 16）
2. LEMS 2 次調査個人票　（添付資料 17）
3. 対応表（LEMS）　（添付資料 18）
4. LEMS 診断基準　（添付資料 5）
5. 情報公開文書　（添付資料 15）

2 次調査終了後（該当施設）

礼状　（添付資料 7）

なお、症例が多い特別階層病院から要望があれば、2 次調査票を Microsoft Excel 入力形式にすることを予定している。

７．観察・検査・報告項目

既にカルテに記録されているデータを集積して解析を行う。項目については、添付書類に記載されている事項となる。

1. MG 2 次調査個人票　（添付書類 9）
2. LEMS 2 次調査個人票　（添付書類 17）

８．有害事象の評価と報告

（１）有害事象の定義と報告方法

■該当なし
□該当あり

（２）研究対象者に生じる負担並びに予測されるリスク及び利益、これらの総合的評価並びに当該負担及びリスクを最小化する対策
■該当なし
□該当あり

９．評価項目
（１）主要評価項目：MG ならびに LEMS の患者数の推計
（２）副次評価項目：MG ならびに LEMS 患者の疫学臨床像の把握

１０．統計的事項
解析項目は協力機関から送られた調査票の回答で、以下の回答用紙に記載された項目
1. 1 次調査票（葉書）　（添付書類 6）
MG ならびに LEMS の患者数推計に使用する

2. MG 2 次調査個人票　（添付資料 9）
3. LEMS 2 次調査個人票　（添付資料 17）
それぞれの疾患において、患者年齢、発症年齢、性別、重症度、検査所見、治療状況、生活状況等を解析する

解析方法
1 次調査：MG ならびに LEMS の別に、診療科・階層毎に、患者あり・なしで分けて整理する。データの集計は、Microsoft Excel を用いて行う。回答された調査票は PDF 化し、研究者間で電子データを共有して、データの入力チェックを行う。

2 次調査：MG ならびに LEMS の別に、各々の個人票に病院番号、診療科番号、通し番号を記入する。データの集計は、Microsoft Excel を用いて行う。回答された調査票は PDF 化し、研究者間で電子データを共有して、データの入力チェックを行う。

１１．症例報告書の記入と報告
症例報告書にあたる 2 次調査票は、協力機関において主治医等が記入する。

１２．倫理的配慮

（１）遵守する倫理指針や法令

「人を対象とする医学系研究に関する倫理指針」（文部科学省・厚生労働省）を遵守する。

（２）個人情報の保護の方法

対象者番号とカルテ番号の対応表は、協力機関が保管して、研究機関は個人を特定することができない。回答された調査票ならびに、それを入力した電子ファイルは、それぞれ施錠された研究室のロッカー、パスワードで保護されたパソコンに保管し，漏洩・盗難・紛失等が起こらないように厳重に管理する。個人情報管理者は足立由美（金沢大学保健管理センター　教授）とする。学会などで研究結果を公表する際には個人が特定できないように配慮し，匿名性を守る。研究の中止又は終了後，学会発表，論文発表のうち，最も遅い時期から，研究に関する電子データ及び実験・観察ノートは 10 年，その他研究データ等は５年保存する。

１３．インフォームド・コンセントを受けるための手続きについて

情報公開によるオプトアウトを行う

情報公開文書（添付資料 15）を研究協力施設に、患者の目に触れるよう掲示を依頼する。

１４．研究対象者に生じる費用負担について

研究対象者に経済的負担は生じない。

１５．本研究に使用する研究費について

難治性疾患等政策研究事業（難治性疾患政策研究事業）（研究課題名）「神経免疫疾患のエビデンスによる診断基準・重症度分類・ガイドラインの妥当性と患者 QOL の検証」（略称「新エビデンス班」）の研究費を用いる。

１６．利益相反について

本研究者の研究担当者は「金沢大学臨床研究利益相反マネージメントポリシー」に従い、金沢大学利益相反委員会に必要事項を申告し、その審査と承認を得るものとする。

１７．実施計画の変更について

研究の進捗にともない、研究内容及び研究組織・期間などに計画の変更の必要が生じた場合は、倫理委員会の承認を得て、変更を行う。

１８．試料・情報について

（１）試料・情報の種類、保存、記録、破棄について

患者診療録、電子カルテ等の診療情報を用いる。

A. 人体から取得した試料

■該当なし

☐該当あり

B. 情報のみ

☐該当なし

■該当あり

情報の種類：通常の診療において取得された診療情報

保存・破棄について：電子データ及び実験・観察ノートは研究終了若しくは中断または，論文等が発表されてから遅い時期から 10 年間，その他の研究データ等は 5 年間保存した後，破棄する。

保存の責任者について：情報は研究責任者　吉川弘明が保管する。

（２）試料・情報の他機関との授受の記録について

【試料・情報を提供する場合（業務の一部委託による提供を含む）】

☐該当なし

■該当あり

・**提供先の機関名称**：

・**提供先の責任者名**：

・**試料・情報**：

1. 金沢医科大学医学部神経内科学
研究責任者：松井　真　（名誉教授、新エビデンス班　前班長）
〒920-0293　石川県河北郡内灘町大学１－１
TEL 076-286-2211　FAX 076-286-3259

2. 自治医科大学公衆衛生学教室
研究責任者：中村好一（教授、厚生労働科学研究費補助金 難治性疾患等政策研究事業（難治性疾患政策研究事業）「難治性疾患の継続的な疫学データの収集・解析に関する研究」研究代表者）
〒329-0498　栃木県下野市薬師寺 3311-1
TEL 0285-58-7338　FAX 0285-44-7217

3. 国際医療福祉大学医学部神経内科
研究責任者：村井弘之（主任教授）
〒286-8686 千葉県成田市公津の杜 4-3
TEL 0476-20-7701

4. 京都府立医科大学大学院医学研究科　地域保健医療疫学教室
研究責任者：栗山長門（准教授）
〒602-8566　京都市上京区河原町通広小路上る梶井町 465 番地
TEL：075-251-5111（代表）FAX：075-211-7093

5. 九州大学大学院医学研究院　成長発達医学（小児科学）
研究責任者：酒井康成（准教授）
〒812-8582 福岡市東区馬出 3-1-1
TEL:092-642-5421

6. 野村芳子　小児神経学クリニック
研究責任者：野村芳子（院長）
〒113-0034 東京都文京区湯島１丁目２番地１３号　お茶の水明神ビル　３階
TEL: 03-3258-5563

7.千葉大学大学院医学研究院　脳神経内科学
研究責任者：桑原　聡　（教授）
〒260-8677 千葉県千葉市中央区亥鼻 1-8-1
TEL: 043-222-7171（代表）

以上の研究機関は共同研究機関である。1 次調査票ならびに 2 次調査票の PDF ファイル画像、Microsoft Excel ファイルに入力したデータを提供し、入力のチェックと解析を共同で行う。

【試料・情報の提供を受ける場合】

□該当なし

■該当あり

共同研究者の国際医療福祉大学医学部神経内科　村井弘之より、2006 年に今回と同様の方法で実施した重症筋無力症の全国疫学調査の匿名化されたデータの提供を受ける。

１９．部局長への報告

- ■ 有害事象報告（随時）
- ■ 試験実施計画書からの重大な逸脱に関する報告（随時）
- ■ 実施状況報告（年１回）
- ■ 終了報告（研究終了時）
- □ その他　（　　　　　）

２０．研究成果の帰属と結果の公表

研究成果から得られた結果は、難治性疾患等政策研究事業（難治性疾患政策研究事業）（研究課題名）「神経免疫疾患のエビデンスによる診断基準・重症度分類・ガイドラインの妥当性と患者 QOL の検証」（略称「新エビデンス班」）に帰属する。
結果は、新エビデンス班の班会議および関連学会で発表されるとともに、関連学術誌に発表される。

２１．研究組織

本学の研究組織
研究代表者：
吉川弘明：　　金沢大学保健管理センター　教授（研究責任者を兼ねる）
研究分担者：
足立由美：　　金沢大学保健管理センター　教授

共同研究機関
1. 金沢医科大学医学部神経内科学
研究責任者：松井　真　（名誉教授、新エビデンス班　前班長）
〒920-0293　石川県河北郡内灘町大学１－１
TEL 076-286-2211　FAX 076-286-3259

2. 自治医科大学公衆衛生学教室
研究責任者：中村好一（教授、厚生労働科学研究費補助金 難治性疾患等政策研究事業（難治性疾患政策研究事業）「難治性疾患の継続的な疫学データの収集・解析に関する研究」研究代表者）
〒329-0498　栃木県下野市薬師寺 3311-1
TEL 0285-58-7338　FAX 0285-44-7217

3. 国際医療福祉大学医学部神経内科

研究責任者：村井弘之（主任教授）
〒286-8686 千葉県成田市公津の杜 4-3
TEL 0476-20-7701

4. 京都府立医科大学大学院医学研究科　地域保健医療疫学教室
研究責任者：栗山長門（准教授）
〒602-8566　京都市上京区河原町通広小路上る梶井町 465 番地
TEL：075-251-5111（代表）FAX：075-211-7093

5. 九州大学大学院医学研究院　成長発達医学（小児科学）
研究責任者：酒井康成（准教授）
〒812-8582 福岡市東区馬出 3-1-1
TEL:092-642-5421

6. 野村芳子　小児神経学クリニック
研究責任者：野村芳子（院長）
〒113-0034 東京都文京区湯島１丁目２番地１３号　お茶の水明神ビル　３階
TEL: 03-3258-5563

7.千葉大学大学院医学研究院　脳神経内科学
研究責任者：桑原　聡　（教授）
〒260-8677 千葉県千葉市中央区亥鼻 1-8-1
TEL: 043-222-7171（代表）

２２．文献

1. Murai H, Yamashita N, Watanabe M, et al. Characteristics of myasthenia gravis according to onset-age: Japanese nationwide survey. J Neurol Sci 2011;305:97-102.
2. 吉川弘明, 潤 清, 荻野美恵子, et al. 重症筋無力症の診断基準の改訂. エビデンスに基づいた神経免疫疾患の早期診断基準・重症度分類・治療アルゴリズムの確立に関する研究　平成 27 年度　総括・分担研究報告書; 2016 3 月: 30-34.
3. 吉川弘明, 潤 清, 荻野美恵子, et al. ランバート・イートン筋無力症候群の診断基準策定. エビデンスに基づいた神経免疫疾患の早期診断基準・重症度分類・治療アルゴリズムの確立に関する研究　平成 27 年度　総括・分担研究報告書; 2016 3 月: 35-37.
4. 中村好一. 難病の患者数と臨床疫学像把握のための 全国疫学調査マニュアル 第 3 版. 難治性疾患の継続的な疫学データの収集・解析に関する研究班　2017 年 1 月

２３．研究に関する業務の一部を委託する場合の、当該業務内容及び委託先の監督方法

■委託しない

□委託する

２４．モニタリングについて

■該当なし

□該当あり

２５．監査について

□該当なし

□該当あり

・・・

その他注意事項
