# Supplementary material for: Comparative study of Japanese nationwide epidemiological studies of myasthenia gravis using datasets of 2006 and 2018
Source: PLoS One. 2025 Oct 9;20(10):e0334041. doi: 10.1371/journal.pone.0334041 (PMC12510604; doi:10.1371/journal.pone.0334041)
Supplement: S3 File — (PDF) [file pone.0334041.s007.pdf]

Reference number: 47-1
Examination number: 2017-292

Research Protocol

1. Assignment name:

National epidemiological survey of myasthenia gravis and Lambert-Eaton myasthenia

2. Overview, purpose, and significance of the research

Countermeasures against intractable diseases in Japan have been carried out in various diseases as research projects to overcome intractable diseases since the Guidelines for Countermeasures against Intractable Diseases in 1972. In immune-mediated neurological diseases, the Immune-mediated Neurological Disease Investigation Research Group worked to elucidate the pathophysiology and etiology and develop treatment methods. Myasthenia gravis (MG) has been investigated and researched as a specific disease since its inception. However, as one of the designated intractable diseases, elucidating its etiology and treatment methods continue to be investigated. National epidemiological surveys on MG were conducted in 1973, 1987, and 2005. Now that more than ten years have passed since the previous survey 1, it seems meaningful to understand the current situation of MG patients in Japan again. In addition, the MG diagnostic criteria were established in 2015 under the Intractable Disease Policy Research Project (Research Project Title) `Establishment of evidence-based early diagnostic criteria, severity classification, and treatment algorithms for neuroimmune diseases. (abbreviated as the Evidence Group), it has been revised based on advances in autoantibody measurement2.

On the other hand, concerning Lambert-Eaton myasthenic syndrome (LEMS), an immune-mediated neurological disease of the neuromuscular junction, no national epidemiological survey has been conducted to estimate the number of patients, and it has not been designated as an intractable disease. The diagnostic criteria for LEMS were established in 2015 as the result of research by the Evidence Group.

It is essential to conduct a national epidemiological survey of MG and LEMS using new diagnostic criteria to deepen our understanding of the epidemiology of these two diseases in our country and to use them to promote evidence-based medical care in the future. In particular, for LEMS, it is necessary to estimate the number of patients through a national epidemiological survey to qualify it as a designated intractable disease. The research plan for this study is to verify the validity of diagnostic criteria, severity classification, guidelines, and patient QOL based on evidence for neuroimmune diseases. (abbreviated as the New Evidence Group) will be carried out as one of the research projects.

A national epidemiological survey of myasthenia gravis was conducted in 2006 using a similar method (research director: Hiroyuki Murai, Department of Neurology, Faculty of Medicine,

International University of Health and Welfare). We received anonymized data from 2006 from Hiroyuki Murai (co-researcher on this study), and we will also conduct a comparative study with the results of this (2018) survey.

3. Scientific rationality and basis of research

We developed a research plan following the National Epidemiological Survey Manual, 3rd Edition for Understanding the Number of Patients with Intractable Diseases and Clinical Epidemiology (January 2017) (Attachment 1). We planned the research following the manual on the continuous collection and analysis of epidemiological data on intractable diseases (representative: Koichi Nakamura), which was supported by the Health, Labor and Welfare Scientific Research Grant-in-Aid for Policy Research on Intractable Diseases (Intractable Disease Policy Research Project)).

4. Research subject selection policy

(1) Eligibility criteria

Following the `National Epidemiological Survey Manual, 3rd edition, to understand the number of patients with intractable diseases and their clinical epidemiology," we mailed request documents and survey forms to medical facilities nationwide.

a) Medical department to be surveyed

The following medical departments were surveyed using the Ministry of Health, Labor and Welfare's Medical Facility Dynamics Survey.

Neurology, internal medicine, pediatrics, respiratory surgery, cardiovascular surgery, surgery, neurosurgery, ophthalmology, otorhinolaryngology

b) Special Tier Hospital

The following hospitals were selected as exceptional and expected to have an exceptionally high concentration of patients.

1. Independent Administrative Institution National Hospital Organization Hokkaido Medical Center
2. Public Interest Incorporated Association Hanamaki General Hospital
3. Neurology Chiba
4. Yoshiko Nomura Pediatric Neurology Clinic
5. Independent Administrative Institution National Hospital Organization Utano Hospital
6. Nagasaki Kawatana Medical Center, National Hospital Organization, Independent Administrative Agency

(2) Exclusion criteria

No setting

5. Target number and research implementation period

(1) Target number

Primary survey: 7,545 institutions (simultaneously surveying MG and LEMS)

Secondary survey: MG=2,000 institutions, LEMS=200 institutions (planned)

Patients to be surveyed shall meet the following conditions.

Primary survey

1. Patients who met the MG diagnostic criteria and were examined during the year 2017 (January 1, 2017, to December 31, 2017) (all cases regardless of whether it was a first visit or a re-examination)
2. Patients who met the LEMS diagnostic criteria and were examined during the year 2017 (January 1, 2017, to December 31, 2017) (all cases regardless of whether it was a first visit or a re-examination)

Secondary survey

1. MG Patients who were confirmed in the last three years (January 1, 2015, to December 31, 2017) among the applicable patients in the primary survey
2. All applicable patients in the LEMS primary survey

6. Research method

Mail the request letter and questionnaire to the selected medical institutions and request them to conduct the survey. Responses will be sent by mail and analyzed after being tabulated. The estimated number of patients will be calculated in the primary survey, and in the secondary survey, a survey will be conducted to clarify the patients' clinical picture.

In the primary survey, a survey on MG and LEMS patients will be conducted simultaneously. The secondary study will investigate MG and LEMS patients separately. The forms required for the investigation are listed below and attached as reference materials.

Primary survey shipping documents Shipping date: March 2018

1. First investigation request letter (Attachment 2)
2. First investigation re-request letter (Attachment 3)
3. MG diagnostic criteria (Attachment 4)
4. LEMS diagnostic criteria (Attachment 5)
5. Primary survey form (postcard) (Attachment 6)

If there are no patients after collecting the primary questionnaire

Thank you letter (Attachment 7)

2nd survey shipping documents Shipping date: September 2018

a) Facility with MG patients
1. MG 2nd investigation request letter (Attachment 8)
2. MG 2nd Survey Individual Form (Attachment 9)
3. Correspondence table (MG) (Attachment 10)
4. MG diagnostic criteria (Attachment 4)
5. Document 1 (MGFA classification) (Attachment 11)
6. Document 2 (MG-ADL scale) (Attachment 12)
7. Document 3 (Masaoka classification) (Attachment 13)
8. Document 4 (mRS) (Attachment 14)
9. Information disclosure document (Attachment 15)

b) Facility with LEMS patients
1. LEMS 2nd Survey Request Letter (Attachment 16)
2. LEMS 2nd Survey Individual Form (Attachment 17)
3. Correspondence table (LEMS) (Attachment 18)
4. LEMS diagnostic criteria (Attachment 5)
5. Information disclosure document (Attachment 15)

After the second survey (applicable facilities)
Thank you letter (Attachment 7)

In addition, if there is a request from a special class hospital with a large number of cases, we plan to make the secondary questionnaire in Microsoft Excel input format.

7. Observation/inspection/report items

Data already recorded in the medical record is collected and analyzed. The items shall be those stated in the attached documents.
1. MG 2nd Survey Individual Form (Attachment 9)
2. LEMS 2nd Survey Individual Form (Attachment 17)

8. Evaluation and reporting of adverse events

(1) Definition and reporting method of adverse events
■Not applicable
□Applicable

(2) The burden on research subjects, the expected risks and benefits, a comprehensive evaluation, and measures to minimize the burden and risk.
■Not applicable
□Applicable

9. Evaluation item
(1) Primary endpoint: Estimation of the number of patients with MG and LEMS
(2) Secondary endpoint: Understanding the epidemiological, clinical picture of MG and LEMS patients

10. Statistical matters
The analysis items are the answers to the questionnaire sent by the cooperating organization and the items listed on the answer sheet below.
1. Primary survey form (postcard) (attached document 6)
Used to estimate the number of patients with MG and LEMS

2. MG 2nd Survey Individual Form (Attachment 9)
3. LEMS 2nd Survey Individual Form (Attachment 17)
For each disease, analyze the patient's age, age of onset, gender, severity, test findings, treatment status, living situation, etc.

Analysis method
Primary survey: Separately, by MG and LEMS, by clinical department and rank, and by whether there are patients. Data aggregation will be performed using Microsoft Excel. The completed questionnaires will be converted into PDF files, the electronic data will be shared among researchers, and data input will be checked.

Secondary survey: Enter the hospital, department, and serial numbers on each form for MG and LEMS. Data aggregation will be performed using Microsoft Excel. The completed questionnaires will be converted into PDF files, the electronic data will be shared among researchers, and data input will be checked.

11. Completing and reporting the case report form

The secondary questionnaire, a case report form, is filled out by the attending physician at the cooperating institution.

12. Ethical considerations

(1) Ethical guidelines and laws to comply with

Comply with the "Ethical Guidelines for Medical Research Involving Human Subjects" (Ministry of Education, Culture, Sports, Science and Technology/Ministry of Health, Labor and Welfare).

(2) How to protect personal information

The collaborating institution will keep the correspondence table between the subject number and medical record number, and the research institution will be unable to identify the individual. The completed questionnaires and the electronic files in which they are entered will be stored in locked laboratory lockers and password-protected computers and will be strictly managed to prevent leakage, theft, or loss. Yumi Adachi (Professor, Kanazawa University Health Management Center) will be the personal information administrator. When publishing research results at academic conferences, etc., prevent individuals from being identified and protect anonymity. After the research is discontinued or completed, electronic data and experiment/observation notes related to the research will be stored for ten years, and other research data will be stored for five years, starting from the latest conference presentations and paper presentations.

13. Procedures for obtaining informed consent

Opt out by disclosing information.
Request the research partner facilities to display the information disclosure document (Attachment 15) so patients can see it.

14. Regarding the cost burden incurred by research subjects

There will be no financial burden on the research subjects.

15. Regarding research funds used for this research

Policy Research Project for Intractable Diseases (Research Project for Intractable Diseases) (Research title) Verification of the validity of diagnostic criteria, severity classification, and guidelines and patient QOL based on evidence for neuroimmune diseases (abbreviation: New Evidence Group)) research funds will be used.

16. Conflict of interest

The person in charge of research of this researcher shall declare the necessary matters to the Kanazawa

University Conflict of Interest Committee and obtain its review and approval following the Kanazawa University Clinical Research Conflict of Interest Management Policy.

17. Regarding changes to the implementation plan

If, as the research progresses, it becomes necessary to make changes to the research content, research organization, period, etc., the changes will be made with the ethics committee's approval.

18. About samples and information

(1) Types of specimens and information, storage, recording, and disposal

Use medical information such as patient medical records and electronic medical records.

A. Samples obtained from the human body

■Not applicable

☐Applicable

B. information only

☐Not applicable

■Applicable

Type of information: Medical information obtained during regular medical treatment

Regarding storage and destruction: Electronic data and experiment/observation notes will be stored for ten years from the end of the research or after the publication of papers, etc., and other research data will be stored for five years and then destroyed.

About the person responsible for preservation: Hiroaki Yoshikawa, the research director, will preserve information.

(2) Records of exchange of specimens and information with other institutions

[When providing samples and information (including provision through partial outsourcing of work)]

☐Not applicable

■Applicable

・**Name of the institution receiving the offer:**

・**Name of the person in charge of the provider:**

・**Sample/Information:**

1. Department of Neurology, Kanazawa Medical University School of Medicine

Research Director: Makoto Matsui (Professor Emeritus, Former Head of the New Evidence Group)

1-1 University, Uchinada-cho, Kahoku-gun, Ishikawa 920-0293

TEL 076-286-2211 FAX 076-286-3259

2. Jichi Medical University Department of Public Health
Research Director: Koichi Nakamura (Professor, Health, Labor and Welfare Grant-in-Aid for Scientific Research, Policy Research on Intractable Diseases (Intractable Disease Policy Research Project) “Research on the continuous collection and analysis of epidemiological data on intractable diseases” Representative)
3311-1 Yakushiji, Shimotsuke City, Tochigi Prefecture 329-0498
TEL 0285-58-7338 FAX 0285-44-7217

3. Department of Neurology, International University of Health and Welfare, School of Medicine
Research Director: Hiroyuki Murai (Chief Professor)
4-3 Kozunomori, Narita City, Chiba Prefecture 286-8686
TEL 0476-20-7701

4. Kyoto Prefectural University of Medicine, Graduate School of Medicine, Department of Community Health and Medical Epidemiology
Research Director: Nagato Kuriyama (Associate Professor)
465 Kajii-cho, Kawaramachi-dori Hirokoji, Kamigyo-ku, Kyoto 602-8566
TEL: 075-251-5111 (main) FAX: 075-211-7093

5. Kyushu University Graduate School of Medicine, Growth and Developmental Medicine (Pediatrics)
Research Director: Yasunari Sakai (Associate Professor)
3-1-1 Umade, Higashi-ku, Fukuoka 812-8582
TEL:092-642-5421

6. Yoshiko Nomura Pediatric Neurology Clinic
Research Director: Yoshiko Nomura (Director)
3rd floor, Ochanomizu Myojin Building, 1-2-13 Yushima, Bunkyo-ku, Tokyo 113-0034
TEL: 03-3258-5563

7. Chiba University Graduate School of Medicine, Department of Neurology
Research Director: Satoshi Kuwabara (Professor)
1-8-1 Inohana, Chuo-ku, Chiba City, Chiba Prefecture 260-8677
TEL: 043-222-7171 (Representative)

The above research institutions are joint research institutions. We will provide PDF images of the primary and secondary questionnaires and data entered into Microsoft Excel files and will jointly check and analyze the input.

[When receiving samples and information]

☐Not applicable

■Applicable

Our co-researcher, Hiroyuki Murai, Department of Neurology, Faculty of Medicine, International University of Health and Welfare, provided anonymized data from a national epidemiological survey of myasthenia gravis conducted in 2006 using a similar method.

19. Report to the department head

■ Adverse event reporting (as needed)

■ Report on significant deviations from the study implementation plan (as needed)

■ Implementation status report (once a year)

■ Completion report (at the end of the research)

☐ Others (   )

20. Attribution of research results and publication of results

The results obtained from the research are based on the Policy Research Project for Intractable Diseases (Research Project for Intractable Diseases) (Research Project Title), Validity of diagnostic criteria, severity classification, and guidelines based on evidence for neuroimmune diseases and patient QOL. Verification (abbreviated as New Evidence Group').

The results will be presented at the New Evidence Group's meetings, academic conferences, and academic journals.

21. Research organization

Research organization of our university

Research representative:

Hiroaki Yoshikawa: Professor, Kanazawa University Health Management Center (also serves as research director)

Co-researcher:

Yumi Adachi: Professor, Kanazawa University Health Management Center

joint research institute

1. Department of Neurology, Kanazawa Medical University School of Medicine

Research Director: Makoto Matsui (Professor Emeritus, Former Head of the New Evidence Group)
1-1 University, Uchinada-cho, Kahoku-gun, Ishikawa 920-0293
TEL 076-286-2211 FAX 076-286-3259

2. Jichi Medical University Department of Public Health
Research Director: Koichi Nakamura (Professor, Health, Labor and Welfare Grant-in-Aid for Scientific Research, Policy Research on Intractable Diseases (Intractable Disease Policy Research Project) Research on the continuous collection and analysis of epidemiological data on intractable diseases Representative)
3311-1 Yakushiji, Shimotsuke City, Tochigi Prefecture 329-0498
TEL 0285-58-7338 FAX 0285-44-7217

3. Department of Neurology, International University of Health and Welfare, School of Medicine
Research Director: Hiroyuki Murai (Chief Professor)
4-3 Kozunomori, Narita City, Chiba Prefecture 286-8686
TEL 0476-20-7701

4. Kyoto Prefectural University of Medicine, Graduate School of Medicine, Department of Community Health and Medical Epidemiology
Research Director: Nagato Kuriyama (Associate Professor)
465 Kajii-cho, Kawaramachi-dori Hirokoji, Kamigyo-ku, Kyoto 602-8566
TEL: 075-251-5111 (primary) FAX: 075-211-7093

5. Kyushu University Graduate School of Medicine, Growth and Developmental Medicine (Pediatrics)
Research Director: Yasunari Sakai (Associate Professor)
3-1-1 Umade, Higashi-ku, Fukuoka 812-8582
TEL:092-642-5421

6. Yoshiko Nomura Pediatric Neurology Clinic
Research Director: Yoshiko Nomura (Director)
3rd floor, Ochanomizu Myojin Building, 1-2-13 Yushima, Bunkyo-ku, Tokyo 113-0034
TEL: 03-3258-5563

7. Chiba University Graduate School of Medicine, Department of Neurology
Research Director: Satoshi Kuwabara (Professor)
1-8-1 Inohana, Chuo-ku, Chiba City, Chiba Prefecture 260-8677

TEL: 043-222-7171 (Representative)

22. Literature

1. Murai H, Yamashita N, Watanabe M, et al. Characteristics of myasthenia gravis according to onset-age: Japanese nationwide survey. J Neurol Sci 2011;305:97-102.
2. Hiroaki Yoshikawa, Kiyoshi Jun, Mieko Ogino, et al. Revision of diagnostic criteria for myasthenia gravis. Research on establishing evidence-based early diagnostic criteria, severity classification, and treatment algorithms for neuroimmune diseases. FY2015 review ・Collaborative research report; March 2016: 30-34. (in Japanese)
3. Hiroaki Yoshikawa, Kiyoshi Jun, Mieko Ogino, et al. Development of diagnostic criteria for Lambert-Eaton myasthenic syndrome. Research establishing evidence-based early diagnostic criteria, severity classification, and treatment algorithms for neuroimmune diseases, FY2015 Summary/shared research report; March 2016: 35-37. (in Japanese)
4. Koichi Nakamura. National Epidemiological Survey Manual for understanding the number of patients and clinical epidemiology of intractable diseases, 3rd edition. Research group on continuous collection and analysis of epidemiological data for intractable diseases January 2017 (in Japanese)

23. When part of research-related work is outsourced, the content of the work and the method of supervising the outsourcing company

■Do not outsource
☐Design

24. About monitoring
■Not applicable
☐Applicable

25. About audit
☐Not applicable
☐Applicable

Other notes
